# A Clinician’s Guide to Topical Retinoids

**DOI:** 10.1177/12034754211035091

**Published:** 2021-07-22

**Authors:** Melika Motamedi, Ahmad Chehade, Ravina Sanghera, Parbeer Grewal

**Affiliations:** 1 3158 University of Alberta, Edmonton, AB, Canada; 2 Division of Dermatology, University of Alberta, Edmonton, AB, Canada; 3 Rejuvenation Dermatology, Edmonton, AB, Canada

**Keywords:** acne, keratinization, retinoid

## Abstract

Retinoids are defined as molecules that bind to and activate retinoic acid receptors to influence the proliferation and differentiation of cells. Topical retinoids have evolved over the past several decades, being used in multiple dermatological conditions. This review aims to differentiate between synthetic and natural retinoids, discuss the pharmacology behind topical retinoids, highlight clinical applications, and categorize all the commercially available agents, including combination products. Understanding retinoid affinities for unique receptor subtypes can impact clinical decisions, resulting in optimizing treatment and enhancing patient adherence.

## Introduction

Topical retinoids have evolved over the past several decades, being used in an array of dermatological conditions. Some of these approved indications include acne vulgaris, psoriasis, photoaging/rhytides, cutaneous T-cell lymphoma, and Kaposi’s sarcoma. They are also used off-label in conditions such as keratosis pilaris and hyperpigmentation.^1^ In general, retinoids are divided into 4 generations based on their molecular structure and receptor selectivity. Topical retinoids are divided into 6 classes. The 6 classes of topical retinoids include: Tretinoin (all-*trans* retinoic acid), adapalene, tazarotene, trifarotene, alitretinoin, and bexarotene. The last 2 classes, alitretinoin and bexarotene, are topical and oral retinoids used in Kaposi’s sarcoma and cutaneous T-cell lymphoma, although infrequently. The availability of alitretinoin and bexarotene topically are limited and are usually required to be compounded. Alitretinoin and bexarotene will not be discussed further in this review. This review aims to differentiate between synthetic and natural topical retinoids, discuss the pharmacology behind topical retinoids, highlight clinical applications, and categorize all the commercially available agents and their combination products.

## The Link Between Vitamin A, Retinoic Acid, and the Body

Retinoids are a class of molecules derived from vitamin A or having structural and/or functional similarities to vitamin A.^2^ Vitamin A is synonymous with retinol; its metabolites include retinaldehyde/retinal and retinoic acid.^3^ This fat-soluble organic compound and its metabolites are involved in immune function, reproduction, vision, cellular communication, and differentiation.^4^ Vitamin A is taken through the human diet in 2 forms, preformed vitamin A (retinol) and provitamin A (carotenoids), and both forms of vitamin A are stored in the liver. Keratinocytes store and convert a majority of vitamin A as retinyl esters in the skin.^5^ Natural topical retinoids commonly used for medical and cosmetic purposes include retinol and the more potent metabolite, retinaldehyde. It should be noted that although retinol can be found in nature from animal and plant sources, most commercially available retinol products are produced synthetically in the lab. Synthetic retinoids, including adapalene, tazarotene, and trifarotene, can interact with the same cellular processes as their naturally occurring counterparts. The skin is a retinoid responsive organ, able to absorb topical retinoids and their derivatives readily. Understanding the biological and cellular pathways of vitamin A involved in the body’s natural processes has allowed researchers to develop treatments targeted towards nuclear receptors involved in this pathway.

## Topical Retinoids Mechanism of Action

Retinoids are defined as a molecule that binds to and activates retinoic acid receptors through direct ligand-receptor binding, thereby eliciting transcription of retinoic acid‐responsive genes.^6^ Retinoids influence the proliferation and differentiation of cells. Their biological effects are mediated and regulated by cytosolic binding proteins and nuclear hormone receptors.^6^ Retinoids normalize abnormal desquamation in acne by increasing follicular epithelial turnover and accelerating the shedding of corneocytes, leading to the expulsion of mature comedones and the suppression of microcomedone formation.^6^

In psoriasis, only the topical retinoid tazarotene is indicated. Tazarotene undergoes hydrolysis in the tissues to tazarotenic acid, which then binds to the retinoic acid receptors.^7^ This receptor-ligand interaction results in the regulation and expression of retinoid-responsive genes, including those involved in cell proliferation and inflammation, a hallmark feature in psoriasis, a condition characterized by increased epidermal proliferation and inflammation.^7^

Tretinoin is the only retinoid with the official indication for photoaging/rhytides. The mechanism by which this occurs is on the molecular level and occurs in 2 different ways, although synergistically.^8^ Tretinoin application before ultraviolet light exposure results in the blocking of activator-protein 1 (AP-1), responsible for the activation of collagen degrading MMP’s, thus inhibiting collagen breakdown.^8^ Additionally, topical application of all-trans retinoic acid induces collagen synthesis by increasing type-1 procollagen expression.^9^

In treating cutaneous T-cell lymphoma, bexarotene, a retinoid selective for the retinoid X receptor (RXR), is indicated.^10^ Bexarotene binds to and activates the RXR nuclear receptors, leading to inhibition of the G1, G2, and *M* phases of the cell cycle, reducing proliferation and increasing apoptosis.^11^

Although retinoids as a class have a similar mechanism, they each still contain unique structures and receptor binding sites, contributing to their differences in indications and effects.

## Pharmacology of Retinoids and Their Receptors

### Nuclear Receptors of Retinoids and Their Roles in Treatment

**Figure 1:**
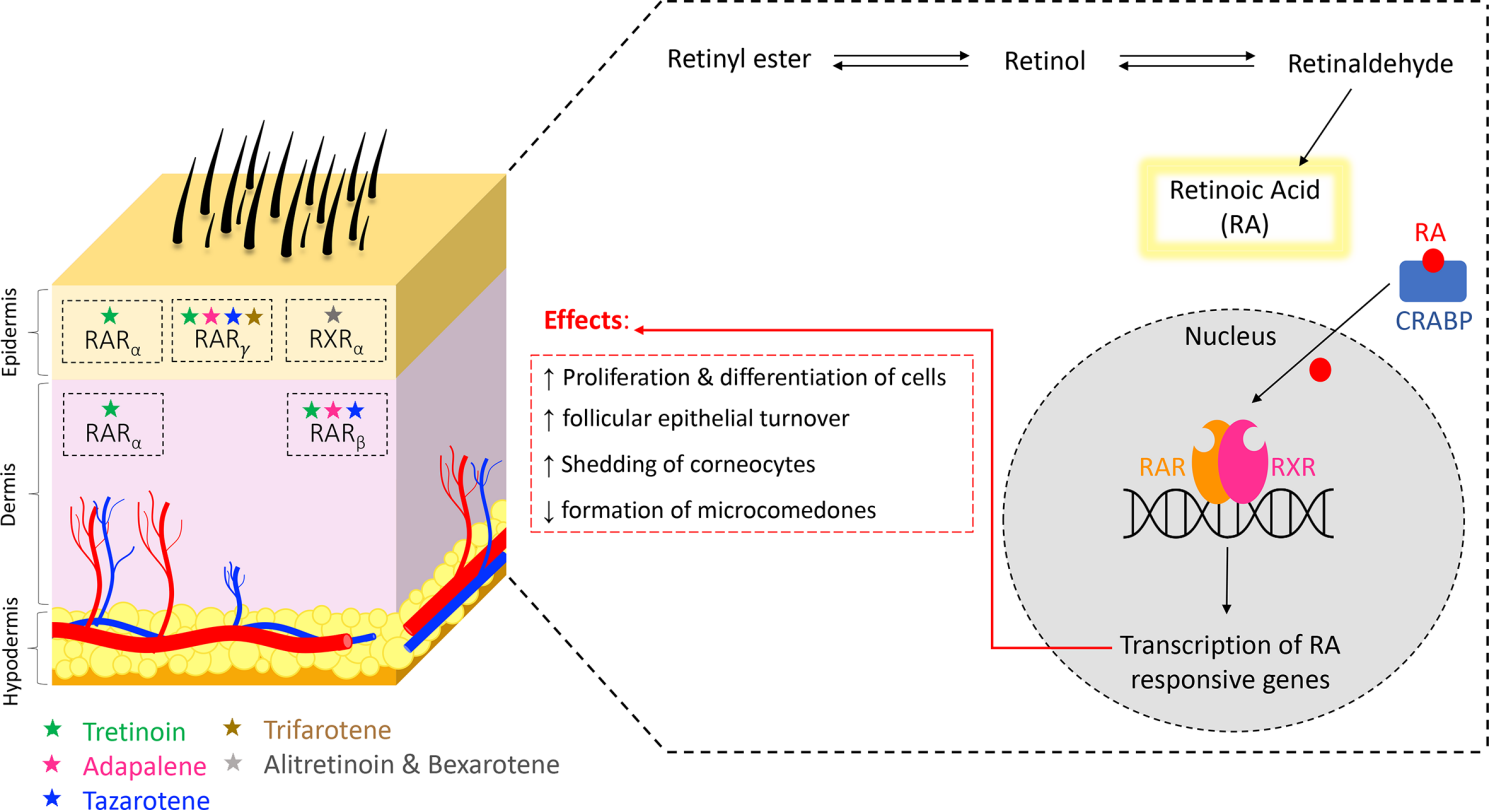
Biological pathway of natural retinoids and target sites of synthetic retinoids.

Retinoic acid receptors (RARs) serve as the binding site for the 2 major natural vitamin-A derivatives, all-*trans* retinoic acid and 9-*cis* retinoic acid.^2^ Naturally, to enter the nucleus, retinoic acid binds to the cytosolic retinoic acid-binding protein (CRABP). It is transported into the nucleus, where the binding of retinoic acid to either RAR or RXR leads to receptor heterodimerization and transcription of various genes (Figure 1). The RARs are also the binding site for synthetic topical retinoids. RXRs belong to the steroid/thyroid hormone receptor family and only bind to the natural vitamin-A derivative, 9-*cis* retinoic acid.^2^ Ligands which only interact with RXRs are referred to as rexinoids. RXRs and Retinoic acid receptors (RARs) are respectively classified as class 1 and 2 nuclear receptors, each of these receptors exhibit ɑ, β, and subtypes.^12^ These 2 receptors exist together as a dimer. RARs heterodimerize with RXRs, while RXRs can homodimerize or heterodimerize with receptors such as the RARs, the vitamin D3 receptor, and the thyroid hormone receptor.^2^ The binding of retinoic acid to the RXR receptor activates the pathway mediated by the receptor that RXR is dimerized with (ex. Vitamin D3).^2^ In this instance, RXR is participating as an active partner within the heterodimer.^2^ Otherwise, RXR participates as a silent partner, where the binding of the RXR receptor by retinoic acid does not influence a response.^2^

In the absence of ligands, dimerized RARs and RXRS are bound to co-repressors.^2^ The presence of corepressors results in chromatin condensation and inaccessible DNA. The binding of natural ligands to these dimers is crucial in the development of various biological processes as it allows for the dissociation of corepressors. These heterodimers are then able to bind specific DNA sequences found within the retinoid-responsive genes, leading to the activation or repression of genes responsible for regulating cell growth, differentiation, and apoptosis.^2,12^

Synthetic retinoids are often prescribed for dermatological conditions. Over the years, these retinoid actions on specific receptor subtypes have been identified to better understand the mechanism and targets of newly developed molecules systemically and topically. However, more understanding of these receptor subtypes, their locations, and the relationship between topical agents and the desired outcomes are still being evaluated.

Receptor subtypes carry different affinities for the various topical retinoids resulting in differences in potency, tolerability, and efficacy of each topical retinoid agent. Receptor subtypes are distributed throughout the various layers of the skin. RAR is found in the epidermis, RARβ is found predominantly in the dermis and other body tissues, and RXRɑ is found throughout all layers of the skin.^13^ The location of these receptor subtypes allows us to understand some of the observed effects of topical agents being used. As demonstrated in Table 1 and Figure 1, tretinoin has a high affinity for all 3 retinoic acid receptor subtypes, whereas adapalene has a selective affinity for RARβ and RAR.^13^ The RAR receptor is associated with terminal differentiation. As abnormal differentiation is a hallmark feature of acne vulgaris, high selectivity for RAR may demonstrate clinical advantages in the treatment of acne.^13^ Although the classes of retinoids commonly used in treating disorders of the skin do not bind to RXR receptors, except for alitretinoin and bexarotene, it is still noteworthy to mention their locations. RXR receptors also include subtypes ɑ, β, and and each RXR has 2 isoforms: RXRα1/α2, RXRβ1/β2, and RXRγ1/γ2.^14^ RXRɑ exists in vital organs of the body, including the liver, lungs, kidneys, and is the primary subtype in the epidermis.^14^ RXRβ is distributed all-over and the body. RXRγ1 is expressed in the brain and muscle, and RXRγ2 is expressed a great deal within skeletal and cardiac muscles.^14^

**Table 1:** Clinically Significant Considerations ^1,26-33,43-47^

Retinoids (monotherapy)	Health Canada indications	Plasma half-life	Ligand-receptor binding sites	Side effects	Contraindications	Pregnancy	Trade name/ Available formulations
Tretinoin (all-trans retinoic acid)	Acne Vulgaris	Normally present in plasma	RAR- ɑ, RAR- β, RAR-	Irritation, local dryness	Hypersensitivity to tretinoin Pregnancy Nursing	Advised not to be used in women pregnant or planning to become pregnant	Stieva-A ® *cream* ***(tretinoin 0.01%, 0.025%, 0.05%)*** Retin-A ® *cream* ***(tretinoin 0.05%)*** Retin-A ® *gel* ***(tretinoin 0.025%)*** Retin-A Micro ® g*el* ***(tretinoin 0.04%, 0.1%)***
Adapalene	Acne Vulgaris	7-51 hours (gel)	RAR- β, RAR-	Irritation, Erythema Peeling of the skin Local dryness	Hypersensitivity Patients with eczema, seborrheic dermatitis Pregnancy/Planning to become pregnant	Category C (Contraindicated in Canada)	Differin ® *gel* ***(adapalene 0.1%,)*** Differin ® c*ream* ***(Adapalene 0.1%,)*** Differin XP ® g*el* ***(Adapalene 0.3%,)***
Tazarotene	Plaque Psoriasis Acne Vulgaris	18 hr (cream, gel)	RAR- β, RAR-	Irritation of skin Local dryness Erythema Pruritus Worsening of psoriasis	Hypersensitivity Pregnancy/Planning to become pregnant	Category X (contraindicated)	Tazorac ® *cream* ***(tazarotene 0.05%, 0.1%)*** Tazorac ® *gel* ***(tazarotene 0.05%, 0.1%)***
Trifarotene	Acne Vulgaris	2-9 hours	RAR-	Irritation of skin Pruritus	Hypersensitivity Patients with eczema, seborrheic dermatitis, Pregnancy/Planning to become pregnant	Contraindicated in Canada	Aklief ® *cream* ***(trifarotene 0.0005%)***

Retinoids (Combo Therapy)	Health Canada Indications	Plasma half-life	Ligand-receptor binding sites -of retinoids	Side effects	Contraindications	Pregnancy category	Trade name/Available formulations
Tretinoin/ Clindamycin	Acne vulgaris	Not available for combo product. Refer to individual agents.	RAR- ɑ, RAR- β, RAR-	Xerosis, pruritus, erythema	In patients with regional enteritis, ulcerative colitis, or history of antibiotic-associated colitis. Patients with a hypersensitivity towards clindamycin, lincomycin, or tretinoin.	Category C	Biacna ® *gel* ***(clindamycin phosphate 1.2%/tretinoin 0.025%)***
Tazarotene/ Halobetasol propionate	Plaque Psoriasis	Not available for combo product. Refer to individual agents. Plasma concentrations were conducted but levels were low following single and repeated application of Duobrii ®	RAR- β, RAR-	Application site reactions including contact dermatitis, folliculitis, and telangiectasia	Patients with hypersensitivity to retinoids or steroids. Patients with fungal, bacterial, or viral skin infections Patients with seborrheic Dermatitis Patients pregnant or planning to become pregnant	Category X	Duobrii ® *lotion* ***(halobetasol propionate 0.01%/ tazarotene 0.045%)***
Adapalene/ Benzoyl peroxide	Acne Vulgaris	Not available for combo products. Refer to individual agents.	RAR- β, RAR-	Application site reactions including erythema, skin irritation, xerosis	Patients with hypersensitivity to retinoids or benzoyl peroxide. Patients with seborrheic Dermatitis or eczema Patients pregnant or planning to become pregnant	Category C (Canadian labeling states that adapalene is contraindicated in pregnant women)	Tactupump ® *gel* ***(adapalene 0.1% /benzoyl peroxide 2.5%)*** Tactupump Forte ® *gel* ***(adapalene 0.3% /benzoyl peroxide 2.5%)***

Natural retinoids are metabolized intracellularly into active retinoic acid (RA), as illustrated in the pathway above. RA then binds to the cytosolic retinoic acid-binding protein (CRABP) and is transported into the nucleus, where the binding of RA to either RAR or RXR leads to receptor heterodimerization and transcription of various genes yielding the listed effects. However, synthetic topical retinoids bind to their specific receptor subtypes indicated above, each one varying in its mechanism of action and metabolic processes.^15,16^

### Retinoids and Pregnancy

Vitamin A (retinol) is essential in various cellular processes and plays a critical role in embryonic development.^17^ Retinoic acid helps regulate embryonic development by activating gene transcription in different locations of the embryo.^18^ Gene knock-out studies in mice have demonstrated that RXR and RAR possession is crucial for embryonic development.^19^ Cells will only respond to retinoic acid if they have the appropriate receptors and if retinoic acid concentrations are maintained at an appropriate range.^19^

The administration of retinoids is contraindicated or advised against in women who are pregnant or planning to become pregnant. In vitro mouse models have demonstrated that retinoids act directly on the embryo causing abnormal development.^19^ This is because developing organs depend on the concentration of accumulated retinoic acid over time (concentration-time relationship) during particular organ development stages. This concentration is influenced by several variables, including the rate at which the maternal intestines absorb retinoids, the plasma half-life of retinoids in maternal plasma, and the rate at which the placenta transfers retinoids from the pregnant mother to the embryo.^20^

Pregnant women may experience a change in their skin during pregnancy. These changes can include an improvement or an exacerbation of pre-existing conditions.^21^ The most common approach in treating acne in pregnancy is utilizing topical therapies, as they are minimally absorbed and have the least chance of affecting the fetus.^22^ Topical retinoids are recommended to be avoided during pregnancy. Tretinoin and adapalene are categorized as category C, and tazarotene is categorized as category X. Both topical tretinoin and adapalene are minimally absorbed, but some studies suggest teratogenicity in the first trimester using these agents.^22^ Studies in the second and third trimester have not shown such a risk, but the concern for systemic effects is raised when large body surface areas are treated, such as in truncal acne or psoriasis.^11,22^ Although there is evidence demonstrating that the risk may be minimal when using agents such as tretinoin, the risk still outweighs the benefits, and all retinoids should be avoided during pregnancy.^23^

Vitamin-A is a normal component of breast milk. Thus we can assume that tretinoin is likely to be excreted in breast milk.^24^ However, the use of topical retinoids and their excretion in breast milk is unknown. Considering the surface area of the body being treated and the agent being considered, it may help guide a clinician’s decision when considering the use of a topical retinoid. With agents such as tretinoin or adapalene, which have conferred some safety data in the later stages of pregnancy, these agents may be better options for clinicians to consider. If topical retinoids are used in breastfeeding, it is recommended to avoid application to large surface areas of the body, specifically with tazarotene, where the application area should be <20% of the body surface area.^24^ Because topical agents can be transferred by direct contact to a breastfeeding infant, mothers should wash their hands following application and avoid direct skin-to-skin contact with the treated areas.^25^

### Clinical Application of Retinoids^26-35^

A clinicians’ decisions to use a topical retinoid is influenced by the condition being treated, a patient’s skin type, previous treatments, and a clinicians comfort with use of a particular agent. In the following discussion, we identify first, second, third, and fourth-generation topical retinoids available to clinicians and their approved indications. Table 1 highlights a summary of the agents, their trade names, and clinically significant considerations.

### First Generation Retinoids

***Tretinoin*** (All-trans retinoic acid) is the first topical retinoid to be developed. Tretinoin is indicated in the treatment of acne vulgaris and photoaging/rhytides. It is also used off-label to treat keratosis pilaris, actinic keratosis, and hyperpigmentation (melasma, solar lentigines).^1^ Tretinoin comes in different formulations, including cream and gel. Tretinoin is one of the more cost-effective retinoids; however, it is slightly irritating and is more photolabile than others.^36^ The development of microsphere technology, seen in tretinoin formulations (Retin-A Micro 0.04%, 0.1%), has helped improve photostability and mitigate some of the adverse effects seen using these agents.^37^ Microspheres help facilitate the delivery of potentially irritating drugs, minimizing irritation, resulting in better patient adherence.^37^

Tretinoin has also been formulated as a combination product with clindamycin for the treatment of acne vulgaris.

### Second Generation Retinoids

There are no topically available second generation formulations of retinoids.

### Third Generation Retinoids

***Tazarotene*** is a topical retinoid indicated in acne vulgaris and the only retinoid indicated in the use of plaque psoriasis. As a mono-therapy, it is available as a cream and gel formulation (0.05% and 0.1%) and is one of the most potent of the retinoids.^38^ It has also been combined in a lotion, with the high potency topical corticosteroid halobetasol, as a topical agent for plaque psoriasis.

***Adapalene*** is a topical retinoid indicated in acne vulgaris. Off-label it is also used in the treatment of hyperpigmentation and actinic keratosis, and due to its tolerability, it is often used off-label for photoaging/rhytides.^1^ It is available in 2 different concentrations, as cream and gel in 0.1% and gel in 0.3% and is available over the counter (OTC) in the United States. Adapalene is the least irritating and least prone to photodegradation,^36^ allowing for daytime application. Adapalene has also been formulated in conjunction with benzoyl peroxide for use in acne vulgaris. It should be noted that both retinoids and benzoyl peroxide can be irritating and drying to the skin; therefore, their use in a combination product can amplify this side effect. Slow titration of combination products used in this class may result in better tolerance over time.

### Fourth Generation Retinoids

***Trifarotene*** is a fourth-generation topical retinoid with selectivity towards the RAR receptor located in the epidermis. Trifarotene is indicated for acne vulgaris of the face and trunk; this agent is available as a cream formulation. It is presumed that the trunk and face indications of this agent are based upon data demonstrating a lower risk of systemic absorption associated with its use. Studies using laboratory testing to assess systemic absorption of trifarotene demonstrated unquantifiable levels within their target populations, those aged (≥18 years) and pediatric patients (9‐17 years) with moderate to severe acne.^39^

## Differentiating Cosmetic Retinols and Prescription Retinoids

Over the years, dermatology practice has taken a larger role in cosmeceuticals. A common topic of discussion amongst patients and practitioners is the distinction between cosmeceutical grade retinoids and prescription-grade retinoids. Cosmeceutical and over the counter retinoids undergo several conversions depending on their initial molecular structure. The conversion sequence is retinyl esters to retinol, which gets converted to retinaldehyde, giving rise to the final product, retinoic acid (Figure 1).^40^ The biologically active form, retinoic acid, is what leads to the improvement in skin texture, fine lines and dyspigmentation.^40^ Since retinaldehyde requires only one conversion step to retinoic acid, compared to 2 steps for retinol, it is considered more potent.

Retinyl esters, retinol and retinaldehyde, which are the 3 precursors to retinoic acid, are classified as cosmeceutical products that can be purchased without a prescription, unlike tretinoin (all-trans retinoic acid) and other prescription retinoids. With respect to choosing a cosmeceutical, the efficacy of retinol is lower in skin treatment which is why retinaldehyde is preferred. In fact it has been shown that most retinoids in cosmeceutical products are deemed ineffective for photoaging unless the ingredient used is retinaldehyde.^41^ Thus, when looking at retinoids for cosmetic purposes, the distinguishing factor between OTC retinoids versus prescription retinoids is their potency. Retinoic acid in its final form can be hundreds of times more potent than cosmetic based retinol or retinaldehyde, resulting in better results and increased side effects, such as erythema, irritation, and dryness.

## Future Developments in Topical Retinoids

Ongoing research in topical retinoids and their receptors will inherently lead to further development of these agents. An area of exploration that has led to novel products in acne treatment is combination therapies with topical retinoids. The utilization of combination therapy with topicals has proven to be advantageous from the perspective of a multimodal mechanism of action and potentially reducing the need for oral treatment and systemic exposure. It also results in improved adherence as patients will only have to adhere to one topical versus 2 separate ones.

Topical retinoids in combination with antibacterial therapy is an avenue that has continuously been explored. The mixture of these 2 agents aid in addressing both inflammatory and non-inflammatory acne, as antibiotics aid in decreasing *C.acne* and dampening inflammation, while retinoids increase cell turnover and aid in comedone exfoliation.^42^ Agents such as tretinoin and clindamycin combinations and adapalene and benzoyl peroxide combinations have proven to be effective. There has been investigation and evaluation in the development of a minocycline and retinoid topical gel.^42^ Studies of this combination demonstrated local delivery of both ingredients and improved clearance of acne-lesions compared to placebo.^42^ These results suggest that there may be a place in treatment with the use of this combination.

Considering the prevalence of acne in youth and adults, it will not be surprising to continue to see new topical retinoids being developed and these agents combined with other proven topical therapies. There is also an interest in formulating agents in new vehicles that help maintain products’ stability and mitigate side effects.

## Conclusion

Topical retinoids have evolved over the decades from first-generation tretinoin, which is still a commonly used treatment approach for many dermatologists. The continued investigation of these agents led to the discovery of third and fourth generation retinoids, which have advantages in potency, tolerability, photostability, and other indications. Research into receptor binding sites of retinoids has also led to discovering a fourth-generation retinoid, trifarotene, which has selectivity towards RAR. Ongoing research will undoubtedly lead to further developments and understanding of topical retinoids and their uses.
